# CK19 and Glypican 3 Expression Profiling in the Prognostic Indication for Patients with HCC after Surgical Resection

**DOI:** 10.1371/journal.pone.0151501

**Published:** 2016-03-15

**Authors:** Jiliang Feng, Ruidong Zhu, Chun Chang, Lu Yu, Fang Cao, Guohua Zhu, Feng Chen, Hui Xia, Fudong Lv, Shijie Zhang, Lin Sun

**Affiliations:** 1 Clinical-Pathology Center, Beijing You-An Hospital, Capital Medical University, Beijing, 100069, China; 2 Surgical Center, Beijing You-An Hospital, Capital Medical University, Beijing, 100069, China; 3 Department of Respiratory Diseases, Peking University Third Hospital, Beijing, 100191, China; 4 Department of Radiology, Beijing You-An Hospital, Capital Medical University, Beijing, 100069, China; 5 The 304th Hospital of PLA, Beijing, 100048, China; 6 Department of Pathology, Beijing You-An Hospital, Capital Medical University, Beijing, 100069, China; Kaohsiung Chang Gung Memorial Hospital, TAIWAN

## Abstract

This retrospective study was designed to investigate the correlation between a novel immunosubtyping method for hepatocellular carcinoma (HCC) and biological behavior of tumor cells. A series of 346 patients, who received hepatectomy at two surgical centers from January 2007 to October 2010, were enrolled in this study. The expressions of cytokeratin 19 (CK19), glypican 3 (GPC3), and CD34 were detected by immunohistochemical staining. The clinical stage was assessed using the sixth edition tumor–node–metastasis (TNM) system (UICC/AJCC, 2010).Vascular invasion comprised both microscopic and macroscopic invasion. The tumor size, lymph node involvement, and metastasis were determined by pathological as well as imaging studies. Recurrence was defined as the appearance of new lesions with radiological features typical of HCC, seen by at least two imaging methods. Survival curves for the patients were plotted using the Kaplan–Meier method, and differences between the curves were assessed using the log-rank test. Significant differences in morphology, histological grading, and TNM staging were observed between groups. Based on the immunohistochemical staining, the enrolled cases were divided into CK19+/GPC3+, CK19−/GPC3+ and CK19−/GPC3− three subtypes. CK19+/GPC3+ HCC has the highest risk of multifocality, microvascular invasion, regional lymph node involvement, and distant metastasis, followed by CK19−/GPC3+ HCC, then CK19−/GPC3−HCC. CK19+/GPC3+ HCC has the shortest recurrence time compared to other immunophenotype HCCs. CK19 and GPC3 expression profiling is an independent prognostic indicator in patients with HCC, and a larger sample size is needed to further investigate the effect of this immunosubtyping model in stratifying the outcome of HCC patients.

## Introduction

Hepatocellular carcinoma (HCC) is the third most deadly malignant tumor in the world. In China, the incidence of this malignancy is approximately 55% of the global incidence. Liver resection is the first-line therapy in patients with solitary tumor and well-preserved liver function. However, high recurrence rate greatly impact the curative effect after hepatectomy. Many independent risk factors, including the tumor size, nodule number, histological grading, and vascular invasion, were identified to be closely associated with the recurrence and survival of HCC patients [1,2]. In the molecule level, the relationship between the expression of some proteins, such as CD133, OV6, CD44, CD47, CK19, and EpCAM, and poorer outcomes of HCC was also established [3–6]. Of note, almost all of these molecules were biomarkers for hepatic stem cell (HSC) or hepatic progenitor cell (HPC).

Traditionally, HCC has long been believed to be transformed from the mature hepatocytes by dedifferentiation process. With the understanding of the hierarchical makeup of parenchymal cells in the adult normal liver or chronic liver diseases, it was recognized that HCC consisted of a heterogeneous group of subtypes, which may have transformed from HPC, as well as the progenies of HPC [7,8]. The relationship between the expression of HSC or HPC biomarkers and poor prognosis of HCC patients prompts us to speculate whether the aggressiveness of tumor cell is associated with the differentiation status of tumor cell before malignant transformation.

To prove the hypothesis, a panel of biomarkers for the distinction of differentiation status of tumor cell is crucial. HPC overexpresses CK19 and possesses a strong bidirectional differentiation potential toward the biliary and hepatocytic lineages. When committed to hepatocytic lineage, HPC decreases the expression of CK19 at the very early stage of differentiation [9–10]. However, cholangiocyte can constantly express CK19. Therefore, CK19 is currently well-accepted as a biomarker for HCC with HPC origin, besides the cholangiocyte carcinoma. According to the published literatures, CK19+ HCC accounts for nearly 20% of all HCCs [3,4].

Fetal protein glypican 3 (GPC3) is another biomarker for HPC, as well as immature hepatocytes. Only at the terminal differentiation period of HPC toward mature hepatocyte, the expression of GPC3 was absent [11,12]. In addition, GPC3 is never expressed in cholangiocyte, cholangiocarcinoma, and low expressed in well-differentiated HCC [13,14]. It was reported that GPC3+ HCC accounts for nearly 72%–81% of all HCCs [15]. According to the specific expression phase and spectrum of CK19 and GPC3 in the differentiation process of HPC toward mature hepatocyte, HCC was subclassified as CK19+/GPC3+, CK19−/GPC3+, and CK19−/GPC3−phenotypes, which roughly corresponded to HCC subtype transform from the HPC, immature hepatocyte, and terminal differentiated hepatocyte, respectively.

Although previous reports have shown the significance of the expression of CK19 [16,17] or GPC3 [18,19] in the prognosis evaluation of HCC patients, in view of the specific expression spectrum of the proteins, combined detection of the two markers can better indicate the differentiation status of tumor cells than utilization of them individually. In the present study, the correlation between the immunosubtyping method of HCC based on the CK19 and GPC3 expression profiling and biological behavior of tumor cells were investigated. Furthermore, the value of this molecular subtyping model in the risk stratification of HCC patients was tested.

## Patients and Methods

The study was approved by the Ethical Committee of Beijing You-An Hospital, Capital Medical University. All methods and procedures associated with this study were accorded ethically with the principles of the Declaration of Helsinki and local laws. All patients provided written informed consent before enrollment. All authors had access to the study data and reviewed and approved the final manuscript.

### Study Design

This was a retrospective study of patients with HCC admitted for hepatectomy. From January 2007 and October 2010, a total of 346 patients, who received a first-time diagnosis of HCC and eventually received pathologic confirmation of the diagnosis, at You-An Hospital, Capital Medical University, and the 304 Hospital, PLA, Beijing, P.R. China, were evaluated.

The entry criteria included the following: (1) the entire tumor (including the main tumor, satellites, and multicenter tumors; at least from the edge of the tumor margins > 1cm) was resectable; (2) liver function was classified as grade A of the Child-Pugh classification; (3) no distant metastases were found (three suspected metastasis cases were identified to be metastases after surgical resection during the follow up); and (4) the remnant volume of the liver was considered adequate and no contraindication to laparotomy was found. The exclusion criteria were as follows: (1) patients with other malignancies; (2) patients with liver function Child–Pugh B and C; (3) patients with less than 1month of life expectancy or follow-up; (4) patients with uncontrolled severe diabetes and acute infection; and (5) patients having no history of chemotherapy or radiotherapy before surgery. Clinical data were extracted from the clinical charts. For comparability among different subtypes of HCC, the clinical stage was assessed using the sixth edition tumor–node–metastasis (TNM) system (UICC/AJCC, 2010) [20]. Vascular invasion comprised both microscopic and macroscopic invasion. The tumor size, lymph node involvement, and metastasis were determined by pathological as well as imaging studies.

### Follow-Up

All patients were followed for 1 month after hepatectomy, then every 3 months during the first year after surgery, and every 6 months thereafter. During follow-up visits, patients were subjected to physical examination, liver function tests, abdominal ultrasonography, and computed tomography or magnetic resonance imaging of the liver. Recurrence was defined as the appearance of new lesions with radiological features typical of HCC, seen by at least two imaging methods. The patients were followed up for at least 36 months. Recurrence-free survival (RFS) was calculated as the time from the date of surgery until the date of tumor recurrence and was censored at the time of last following-up or death if at that time there was no evidence of tumor recurrence.

### Hematoxylin–Eosin (HE) and Immunohistochemical Stains

Routine HE staining and immunohistochemistry staining were performed as described in a previous study [21]. Mouse anti-human CK19 monoclonal antibody (Clone BA17; dilution, 1:100) and Mouse anti-human GPC3 monoclonal antibody (Clone 1G12; dilution, 1:200) were purchased from Zeta Corporation (Sierra Madre, CA, USA). Mouse anti-human monoclonal antibody CD34 (Clone QBEnd/10; dilution, 1:100) was purchased from Zymed Laboratories (San Francisco, CA, USA). Steamer for 20 minutes in citrate target retrieval buffer (pH 6.0). The results of immunohistochemical staining were considered positive if greater than 10% of the tumor cells showed cytoplasmic staining for CK19, GPC3, or CD34. Probably due to the spontaneous differentiation, in some of the CK19 or GPC3 positive HCC, patchy positive for CK19 or GPC3 staining in varying degrees could be observed. In these cases, when the tumor cells co-expression CK19 and GPC3 (more than 5%), they were classified into the CK19+/GPC3+ group [3,19]. Histologically, all of the primary carcinoma of the liver with CK19+/GPC3− phenotype in our center meets the diagnostic criteria of the intrahepatic cholangiocarcinoma, which were excluded from current analyses. Therefore, all enrolled cases were divided into three groups: CK19+/GPC3+ group, included cases where tumor cells coexpress CK19 and GPC3; CK19−/GPC3+ group, included cases where tumor cells express GPC3 singly; and CK19−/GPC3− group, included cases with negative expression of both CK19 and GPC3. The evidence of cytoplasmic staining of adjacent interlobular duct epithelia served as internal positive control for CK19; yolk sac tumor tissue was used as a positive control sample for GPC3. Negative controls were carried out by substitution of the primary antibodies with nonimmunized serum, which resulted in no signal detection. The expression of these markers was assessed independently and blindly by two consultant histopathologists (JF and FL). All slides were reviewed to confirm the diagnosis according to the guidelines of the World Health Organization (WHO) criteria, 2010 [20]. CD34 staining was helpful in the recognition of a trabecular pattern.

### Statistical Analysis

All analyses were performed using the SPSS 13.0 software (SPSS Inc., IL, USA). The data were expressed as mean± standard deviation. The chi-square test and Student *t*-test were applied to compare the distribution of categorical and continuous variables between the groups, respectively. Survival curves for the patients were plotted using the Kaplan–Meier method and differences between the curves were assessed using the log-rank test. The Cochran–Armitage trend test was used to study the underlying trend. Linear regression with collinearity diagnostics was performed for data correlated with RFS. A variance inflation factor (VIF) higher than 10 was indicative of high collinearity. Univariable and multivariable Cox proportional hazards regression analysis was used to assess factors associated with recurrence of HCC. *P*< 0.05 was considered statistically significant.

## Results

During the study period, 422 HCC patients were treated at the two hospitals. Of these, 63 (14.93%) were excluded because they had received local ablation therapy, ethanol injection, or transarterial chemoembolization before the hepatectomy. And, another 13 (3.08%) were excluded because they did not participate in the follow-up. The remaining 346 (81.99%) patients satisfied the inclusion criteria and were included in the present study. Of these, 121 (34.97%), 47(13.58%), and 20 (5.78%) experienced recurrence within 1, 2, and 3 years after hepatectomy, respectively. The other 19(5.49%) experienced recurrence beyond 3 years after hepatectomy. The IHC staining showed 69 CK19+/GPC3+ cases, 224 CK19−/GPC3+ cases, and 53 CK19−/GPC3− cases accounting for 19.94%, 64.74%, and 15.32% of all enrolled patients, respectively.

### Baseline Characteristics

As shown in S1 Table, the average onset age of CK19+/GPC3+ HCC was earlier than that of the other subtypes, but difference between CK19−/GPC3+ and CK19−/GPC3− HCC was not significant. All HCCs were more common in males than females. A significant difference in gender distribution was not found between any two HCC subtypes. Significant differences in underlying cirrhosis between any two HCC subtypes were not found.

### Relationship between Immunosubtype and Histological Features of HCC

#### Correlation between immunosubtype and morphological classification or histological grading of HCC

Morphologically, acinar/thin trabecular HCC (57/346), thick trabecular HCC (243/346), and compact variants (12/346) were predominant in the present series. Pseudoglandular variant usually present as a component of thin trabecular HCC, therefore they were combined into the same group. Thirty-four scirrhous HCCs (SHCCs), a subtype of HCC characterized by diffused fibrosis along the sinusoid-like blood spaces, were found in the present series [22]. Majority of SHCCs (31/34) showed CK19+/GPC3+ expression. Thirty-three CK19+/GPC3+cases (33/69) with thick trabecular structure also showed abundant fibrous stroma. According to the parenchymal cell to fibrous stroma ratio, they do not fully meet the diagnostic criteria of SHCC. However, fibrous stroma among the tumor nests was very rich compared to that in CK19−/GPC3+ and CK19−/GPC3− groups.

**Fig 1:**
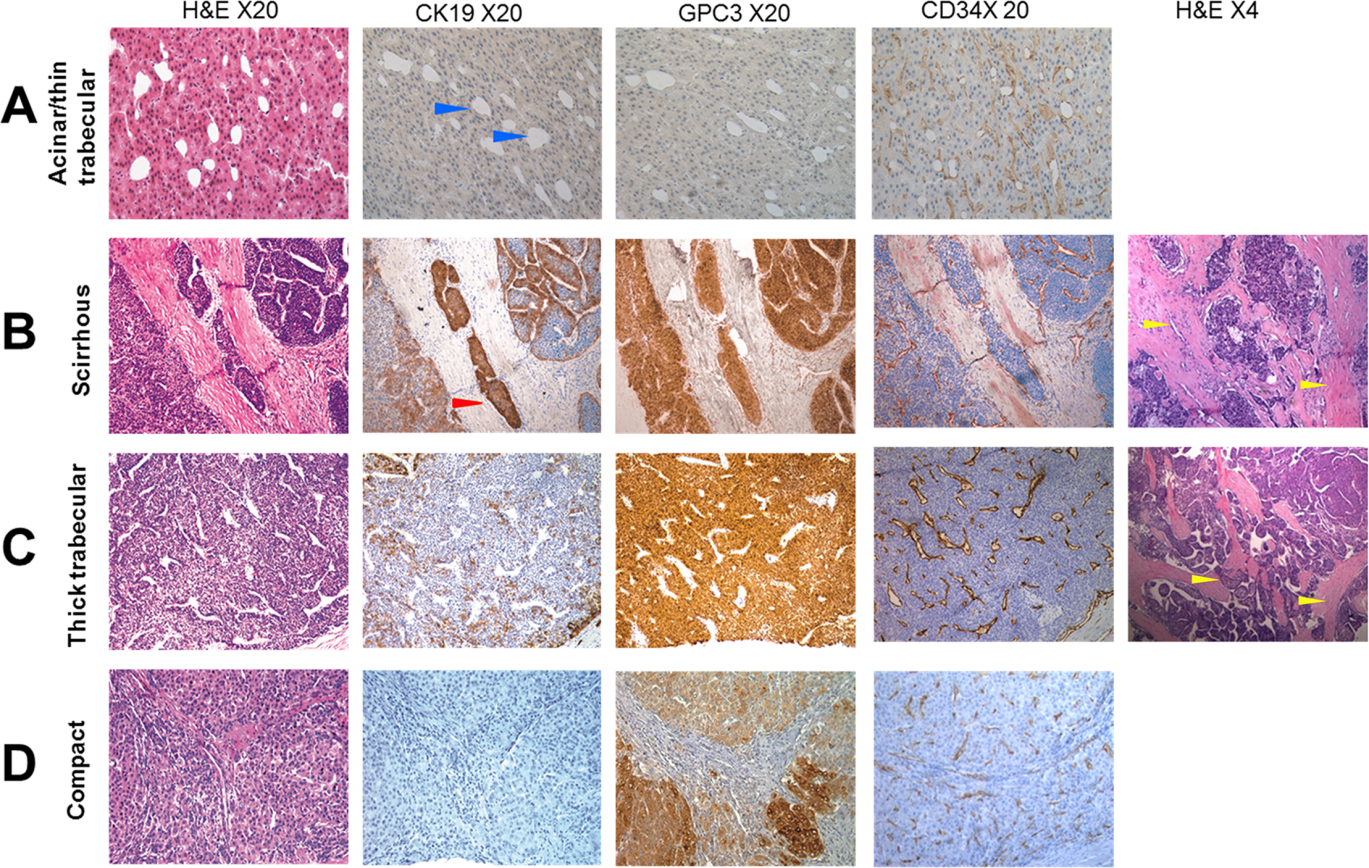
CK19 and GPC3 expression in different histological variation of HCC. Thin trabecular HCC was usually accompanied by the pseudoglandular structure (blue arrow head). This histological variant mostly showed CK19−/GPC3− expression (A). Majority of SHCCs showed CK19+/GPC3+ expression. But nearly half of CK19+/GPC3+ cases with thick trabecular structure also showed abundant fibrous stroma. According to the parenchymal cell to fibrous stroma ratio, they do not fully meet the diagnostic criteria of SHCC (B, C). Most of the compact form of HCC present with CK19−/GPC3+ expression. CD34 staining showed that sinusoid-like blood spaces were not abundant compared to other histological variants (D).

Thick trabecular and scirrhous variants accounted for 47.83% (33/69) and 44.93% (31/69) of all CK19+/GPC3+ cases, respectively. Compared to that of the acinar/thin trabecular and compact variants, the difference in the distribution of the thick trabecular or scirrhous variants in CK19+/GPC3+ HCC was significant (*P*<0.01; *P*<0.01). Tumors with thick trabecular pattern accounted for 91.96% (206/224) of all CK19−/GPC3+ HCC, which was significantly higher than that of other histological variants in the CK19−/GPC3+ group (*P*<0.01). Acinar/thin trabecular variants accounted for 83.02% (44/57) of all CK19−/GPC3− HCC cases, which was significantly higher than that of the other histological variants in the CK19−/GPC3−group (*P*<0.01). These results showed close correlation between the immunosubtype and morphological classification of HCC. CK19+/GPC3+ HCCs were more prone to present with thick trabecular pattern accompanied by fibrous stroma. CK19−/GPC3− HCCs more likely appeared in the acinar/thin trabecular pattern, which mimicked the normal hepatic plates. And CK19−/GPC3+ HCCs more likely presented in the thick trabecular pattern (Table 1; Fig 1).

**Table 1:** The relationship between the histological variation and immune-subtypes of HCC.

Histological variation	CK19+/GPC3+	CK19-/GPC3+	CK19-/GPC3-
Acinar/thin trabecular	4 (5.80)	9 (4.02)	44 (83.02)
Thick trabecular	33 (47.83)	206 (91.96)	4 (7.55)
Compact	1 (1.45)	8 (3.57)	3 (5.66)
Scirrhous	31 (44.93)	1 (0.41)	2 (3.77)
n	69	224	53
Χ^2^	523.31	159.46	206.45
P	P<0.01	P<0.01	P<0.01

Data are given as number (percentage). HCC, hepatocellular carcinoma; CK19, cytokeratin 19; GPC3, glypican 3

Well-differentiated HCC usually presented in the thin trabecular pattern (1–3 hepatocytes thick) and minimal nuclear atypia. CK19−/GPC3− and CK19−/GPC3+cases accounted for about 59.09% and 40.91% of all well-differentiated HCC patients, respectively. No CK19+/GPC3+ cases presented in the well-differentiated HCC group. Compared to that in the moderately or poorly differentiated HCC, lower percentage of CK19−/GPC3+ cases in well-differentiated HCC were found (*P*<0.01; *P*<0.01). Compared to that in the CK19−/GPC3+or CK19+/GPC3+ groups, the percentage of poorly differentiated cases were significantly lower in the CK19−/GPC3− group (*P*<0.01; *P*<0.01). The tendencies were observed in the distribution of the well- or poorly differentiated HCC groups along with the CK19+/GPC3+, CK19−/GPC3+, CK19−/GPC3− phenotype (*P*_trend_<0.01; *P*_trend_<0.01). However, the difference in the distribution of moderately differentiated HCC between any two immunophenotype HCC was not significant (*P*>0.05; *P*>0.05; *P*>0.05) (Table 2).

**Table 2:** The correlation between immune-phenotype and histological grading of HCC.

Immuno-pehnotype	Well	Moderately	Poorly	n
CK19+/GPC3+	0 (0.00)	26 (37.68)	43 (62.32)	69
CK19-/GPC3+	9 (4.02)	102 (45.54)	113 (50.45)	224
CK19-/GPC3-	13 (24.53)	25 (47.17)	15 (28.30)	53
CK19+/GPC3+ *vs*. CK19-/GPC3+	Χ^2^ = 1.67 P>0.05	Χ^2^ = 1.32 P>0.05	Χ^2^ = 2.99 P>0.05	
CK19+/GPC3+ *vs*. CK19-/GPC3-	Χ^2^ = 18.94 P<0.01	Χ^2^ = 1.11 P>0.05	Χ^2^ = 13.91 P<0.01	
CK19-/GPC3+ *vs*. CK19-/GPC3-	Χ^2^ = 21.93 P<0.01	Χ^2^ = 0.05 P>0.05	Χ^2^ = 8.46 P<0.01	

Data are given as number (percentage). HCC, hepatocellular carcinoma; CK19, cytokeratin 19; GPC3, glypican 3

#### Multicollinearity analysis

Since the regression coefficients may be compromised by collinearity, the VIF was checked as an indicator for collinearity. Linear regression with collinearity diagnostics showed collinearity didn’t exist within the immunophenotype, histological classification, and grading degree of HCC (VIF = 1.097).Therefore, these correlated factors can be regarded as the major factors.

### Relationship between the immunosubtype and TNM or clinical staging of HCC

As shown in Table 3, in stage I HCC, patients with CK19−/GPC3− expression took up the highest percentage (49.06%), then CK19−/GPC3+(41.07%) and CK19+/GPC3+(17.39%) (0.01<*P*<0.05). However, in stage IV HCC, the result was just the opposite (*P*<0.01). The difference in the distribution of the three immunophenotype HCC in stage II or III group was not statistically significant.

**Table 3:** Correlation between the immune-phenotype and clinical staging of HCC.

Immuno-phenotype	I	II	III	IV	n	Χ^2^	P
CK19+/GPC3+	12 (17.39 9.23)	26 (37.68 21.85)	23 (33.33 26.74)	8 (11.59 72.73)	69	17.50	P<0.01
CK19-/GPC3+	92 (41.07 70.77)	76 (33.93 63.87)	53 (23.66 61.63)	3 (1.34 27.27)	224	2.39	P>0.05
CK19-/GPC3-	26 (49.06 20.00)	17 (32.08 14.29)	10 (18.87 11.63)	0 (0.00 0.00)	53	3.99	P>0.05
n	130	119	86	11	346		
Χ^2^	8.14	0.23	2.24	17.81			
P	0.01<P<0.05	P>0.05	P>0.05	P<0.01			

Data are given as number (percentage). HCC, hepatocellular carcinoma; CK19, cytokeratin 19; GPC3, glypican 3

The tendencies in the increase of the percentage of CK19+/GPC3+ cases and decrease of the percentage of CK19+/GPC3+ and CK19−/GPC3− cases along with the clinical staging from I to IV were also observed (*P*_trend_<0.01; *P*_trend_<0.05; *P*_trend_<0.05).

As shown in Table 4, the differences in multifocality, microvascular invasion, regional lymph node involvement, and distant metastasis between CK19+/GPC3+ and CK19−/GPC3+ or CK19−/GPC3− groups were significant. In addition, the difference in microvascular invasion between CK19−/GPC3+ and CK19−/GPC3− groups was also significant. The Cochran–Armitage trend test showed that the rates of multifocality, microvascular invasion, regional lymph node involvement, and distant metastasis increased significantly along with the alteration of phenotype from CK19−/GPC3− to CK19−/GPC3+, then CK19+/GPC3+ (*P*_trend_<0.01; *P*_trend_<0.01; *P*_trend_<0.01; *P*_trend_<0.01). These results showed that the malignant behavior of HCCs was closely associated with their immunophenotype.CK19+/GPC3+ HCC was the most aggressive subtype, followed by the CK19−/GPC3+ HCC. CK19−/GPC3− HCC was the least aggressive subtype.

**Table 4:** The correlation in TNM staging between any two immune- subtype HCC.

Phenotype	Multifocality	Microvascular Invasion	Involvement of major branch of vein	Liver capsule invasion	Regional lymph node involvement	Distant metastasis	*n*
CK19+/GPC3+	31(44.92)	43(62.32)	7(10.14)	3(4.34)	7(10.14)	8(11.59)	69
CK19-/GPC3+	56(25.00)	101(45.08)	10(4.46)	18(8.03)	7(3.12)	3(1.33)	224
CK19-/GPC3-	8(15.09)	13(24.52)	3(5.66)	5(9.43)	0(0.00)	0(0.00)	53
CK19+/GPC3+ *vs*. CK19-/GPC3+	Χ^2^ = 10.03 P<0.01	Χ^2^ = 6.27 0.01<P<0.05	Χ^2^ = 2.16 P>0.05	Χ^2^ = 0.60 P>0.05	Χ^2^ = 4.27 0.01<P<0.05	Χ^2^ = 12.65 P<0.01	
CK19+/GPC3+ *vs*. CK19-/GPC3	Χ^2^ = 12.27 P<0.01	Χ^2^ = 17.24 P<0.01	Χ^2^ = 0.32 P>0.05	Χ^2^ = 0.57 P>0.05	Χ^2^ = 3.98 0.01<P<0.05	Χ^2^ = 4.82 0.01<P<0.05	
CK19-/GPC3+ *vs*. CK19-/GPC3-	Χ^2^ = 2.37 P>0.05	Χ^2^ = 7.48 P<0.01	Χ^2^ = 0.00 P>0.05	Χ^2^ = 0.00 P>0.05	Χ^2^ = 0.67 P>0.05	Χ^2^ = 0.01 P>0.05	

Data are given as number (percentage). TNM, the TNM (Tumor, Node, Metastasis) staging system; CK19:cytokeratin 19; GPC3:glypcan3

### Univariate Analysis with Respect to Tumor Recurrence

The total recurrence rate of patients was 59.8%. Median time to recurrence was 23 months. RFS was compared for 11 possible prognostic factors, including age, gender, presence of cirrhosis, multiplicity of tumors, greatest tumor dimension, immunophenotype, microvascular invasion, involvement of major branch of vein, perforation of visceral peritoneum, regional lymph node involvement, distant metastasis, histological grading, and the American Joint Committee on Cancer (AJCC) clinical staging. Using the Kaplan–Meier method, univariate analysis showed that multiplicity, microvascular invasion, macrovascular invasion, histological grading, immunophenotype, histological grading, AJCC clinical staging, and regional lymph node involvement were significantly associated with the RFS of patients (Table 5).

**Table 5:** Univariate analysis with respect to tumor recurrent.

		Survival				P value
Chararistic	n	6nonth	12nonth	24month	36month	
Gender						P = 0.155
Male	283	76.210	63.269	45.593	34.178	
Female	63	87.004	70.124	53.601	50.218	
Age						P = 0.0975
<50	125	77.033	57.925	39.091	27.567	
>50	211	82.203	68.271	51.443	42.079	
Cirrhosis						P = 0.2238
Yes	273	76.798	62.906	44.399	37.043	
No	73	83.349	70.635	47.341	36.946	
Size of main nodule (cm)						P = 0.1323
<3	171	82.981	66.315	48.815	36.867	
3<D<5	72	77.512	70.466	53.607	41.755	
>5	103	70.515	57.260	38.914	33.712	
Nodule number						P<0.0001
1	251	82.411	70.739	52.350	41.609	
1<n≤3	95	66.714	47.549	32.107	23.711	
Microvascular invasion						P<0.0001
Yes	158	65.373	50.707	32.472	25.215	
No	188	88.794	75.927	58.369	46.192	
Macrovascular invasion						P = 0.0014
Yes	20	58.438	31.875	15.938	15.938	
No	326	79.323	66.449	48.853	38.098	
Histological grading						P = 0.0002
Poorly	171	68.034	55.260	35.175	28.341	
Moderately	153	87.532	73.589	55.124	42.824	
Well	22	90.911	72.727	72.727	57.132	
Immuno-phenotype						P<0.0001
CK19+/GPC3+	69	57.155	44.494	24.235	16.524	
CK19-/GPC3+	224	82.927	68.818	52.103	40.460	
CK19-/GPC3-	53	84.615	69.127	57.937	49.354	
Clinical staging (6th AJCC)						P<0.0001
I	130	90.000	77.685	66.615	52.891	
II	119	78.047	62.359	34.100	22.627	
III	86	60.986	47.569	31.443	28.242	
IV	11	71.616	61.364	51.124	40.921	
Perforation of visceral peritoneum						P = 0.7130
Yes	26	80.841	65.385	45.313	45.313	
No	320	77.939	64.450	47.067	36.437	
Regional lymph node involvement						P = 0.0140
Yes	14	41.734	33.317	33.317	25.013	
No	332	79.687	65.820	50.619	37.355	
Distant metastasis						P = 0.4437
Yes	14	77.938	62.338	38.961	38.961	
No	332	78.177	64.617	47.439	36.638	

CK19:cytokeratin 19; GPC3:glypcan3; AJCC: American Joint Committee on Cancer

**Fig 2:**
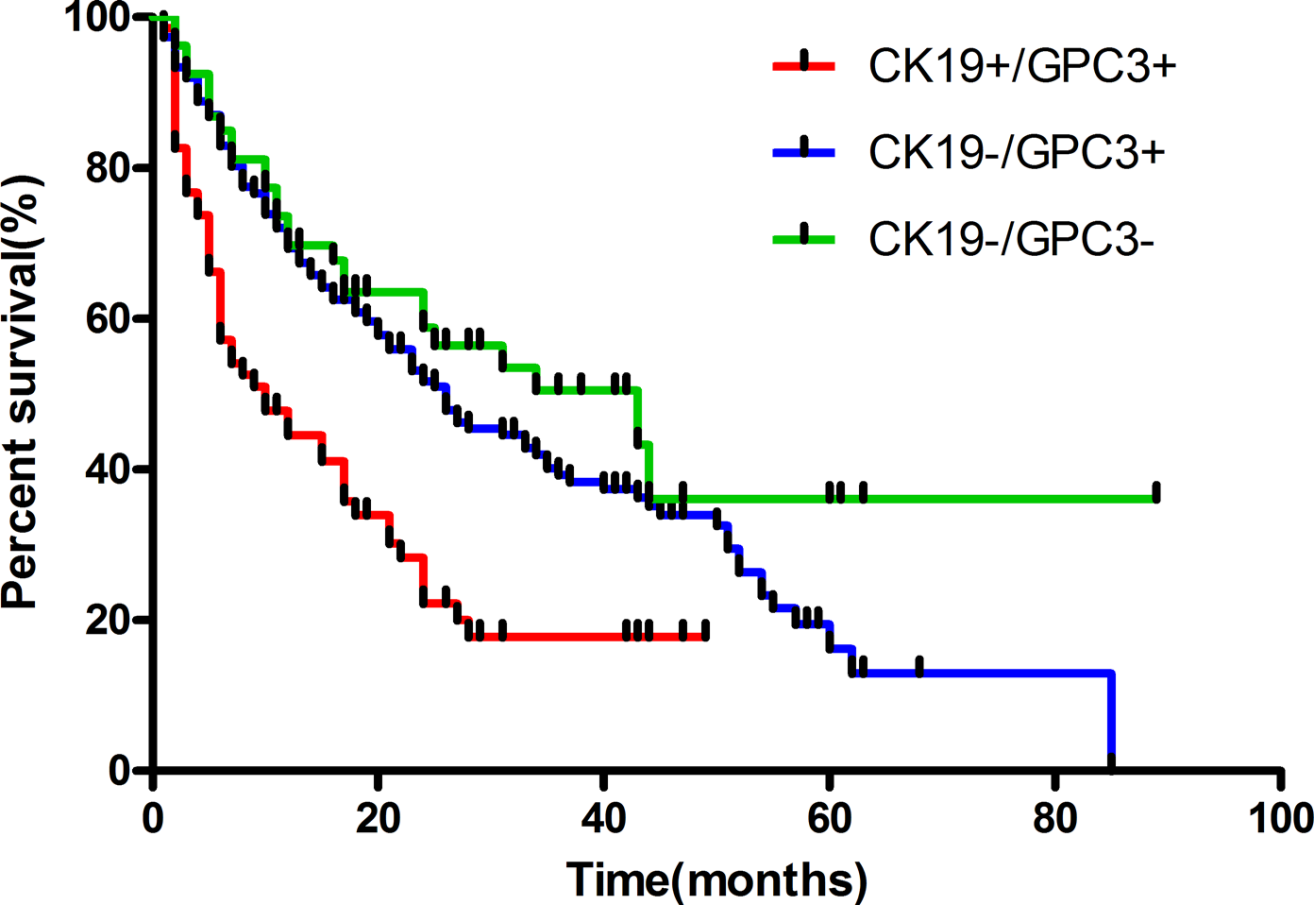
The log-rank test showed a significant difference among the survival curves of three immunosubtypes of HCC (log-rank statistic = 22.61, d.f. = 2, *P* < 0.01).

Median time to recurrence of CK19+/GPC3+, CK19−/GPC3+, and CK19−/GPC3− groups was 10, 26, and 43 months, respectively. The corresponding 1-, 2-, and 3-year survivals were 44.49%, 24.24%, and 16.52%; 68.82%, 52.10%, and 40.46%; and 69.13%, 57.94%, and 49.35%, respectively. The log-rank test showed a significant difference among the survival curves of the three subtypes (*P*<0.001).The cumulative RFS rate was significantly higher in CK19−/GPC3+ and CK19−/GPC3− groups than in the CK19+/GPC3+ group (*P*<0.01; *P*<0.01).However, the cumulative RFS rate was similar between CK19−/GPC3+ and CK19−/GPC3− groups (*P*>0.05) (Fig 2).

### Multivariable Cox Proportional Hazards Regression Analysis of Risk Factors Associated with Recurrence

The seven factors that were significant in the univariate analysis were entered in the multivariate analysis as shown in Table 6. Only immunophenotype [hazard ratio (HR) = 0.709, 95%confidence interval (CI) = 0.545–0.921, *P* = 0.01], histological grading (HR = 0.740, 95%CI = 0.569–0.962,*P*<0.05), multiplicity of tumors (HR = 1.572, 95%CI = 1.137–2.174, *P*<0.01), and microvascular invasion (HR = 1.509, 95%CI = 1.074–2.120, *P*<0.05) were shown to be independent predictors of RFS.

**Table 6:** Multivariable Cox proportional hazards regression analysis.

Chararistic	HR	95.0% CI	P value
Immuno-phenotype	0.709	0.545–0.921	0.010
Histological grading	0.740	0.569–0.962	0.025
Nodule number	1.572	1.137–2.174	0.006
Microvascular invasion	1.509	1.074–2.120	0.018
Macrovascular invasion	1.352	0.790–2.314	0.271
Clinical staging	1.000	0.813–1.230	0.999
Regional lymph node involvement	1.571	0.809–3.050	0.182

HR: hazard ratio; CI: Confidence Intervals

## Discussion

In the present study, it was found that from CK19+/GPC3+ HCC to CK19−/GPC3+ HCC, and then CK19−/GPC3− HCC, tumor cell in intrahepatic metastasis, microvascular invasion, regional lymph node involvement, and distant metastasis was decreased. In addition, the multivariate analysis showed that this immune subclassification method of HCC was an independent predictor of RFS. These evidences supported that the cellular differentiation status was closely linked to the aggressive potential of the tumor. Of note, the term “differentiation” in this study was restored to its meaning in developmental biology, which is distinct from the same term used commonly in the oncology research field to describe the histological grading degrees based on the morphological observation. Although closely correlated, collinearity diagnosis showed no collinearity problem between the two factors, which indicated that both cellular differentiation status and histological grading were independent predictors of RFS.

Edmondson-Steiner grading system is the most commonly used method for the histological grading of HCC. In this system, cellular and architectural atypia is the crucial criteria. Since tumorigenesis and progression were principally a consequence of accumulated mutations, a striking atypia of tumor means more accumulated mutations a tumor acquired [23–25], which can be the reason for the correlation between histological grading and HCC recurrence. However, this genetic alteration mechanism cannot fully explain why the aggressive behavior of tumor cells is closely associated with the cellular status of differentiation in HCC.

In normal development, organogenesis, and tissue repair, the whole human genes are well-programmed, subtly modulated, and dynamically expressed, which is beneficial to the development of body and maintenance of organ function. Compared to the terminal differentiated or immature cells, the genetic and epigenetic expression profile in stem or undifferentiated cell is greatly different, which determines the alteration of biological properties and functions, such as cellular adhesion and migration capacities from stem cells to terminal differentiated cells [26,27]. Under carcinogenic stimuli, quite a lot of genetic or epigenetic processes in tumor initiating cells can be altered; however, there still remain an overwhelming majority of normal genetic and epigenetic processes that are not involved and continue into the transformed cells [28–30]. Although these normal events may not be contributors for the onset of malignant transformation, yet could be preserved by the transformed cells and present in an aggressive profile, such as invasion and metastasis. This might be the reason why HCCs with primitive immunophenotype of differentiation are more aggressive.

The current work showed that the multiplicity of tumors and microvascular invasion were the independent predictors of RFS for patients with HCCs, which was consistent with several other studies [31]. Multifocal HCC can be of multicentric origin or intrahepatic metastases arising from a primary HCC. Clonal analysis from several independent studies showed that 64%–74.2% of multiple nodules in HCC was intrahepatic metastasis [31–33], which implied, to some degree, the presence of multiplicity under the same background just reflects a stronger invasive capacity of tumor cells. In addition, microvascular invasion can be regarded as essentially intrahepatic metastases, the presence of which also indicates a stronger invasive capacity of tumor cells. Therefore, multiplicity of intrahepatic metastasis, microvascular invasion, regional lymph node involvement, and distant metastasis were just the consequences of aggressive biology of tumor cells. Since the cellular biological property provides intrinsic impetus for the aggressive behavior of tumor cells and information closely linked to the tumor biology provides the most crucial evidence for the assessment of malignancy risk of tumors, compared to the cellular differentiation status and histological grading, the value of multiplicity of intrahepatic metastasis and microvascular invasion in clinical assessment and prognostic evaluation for tumors at early stages is limited.

Currently, the use of some omic techniques has empowered the identification of prognostic subclasses in a wide variety of tumors. In these studies, data retrieved from the omic approaches were all based on the preclassification of patients according to the different clinical outcomes or histological grading degrees of tumors [34]. In the liver, several classification and prognostic predicting systems for HCC were put forward [35–37]. However, they are scarcely used in oncology practice, indicating that these molecular prognostic or predictive tests for HCC have not yet achieved their full potential.

This study has some limitations. The main limitation of this study was the small sample size, particularly the small number of CK19−/GPC3− patients, which resulted in very low power to detect differences in the cumulative RFS rate between the CK19−/GPC3− and CK19−/GPC3+ group patients. In addition, this was a retrospective study conducted at only two centers. A standardized multicenter collaborative study is still needed to highlight the importance of this immuneclassification model in the risk stratification of HCC patients and generate high-level medical evidence so that a uniform set of criteria can be established and recognized.

## Conclusions

With the combined detection of CK19 and GPC3, the significance of differentiation status of tumor cells was first revealed in the assessment of aggressive biological behavior of tumor cell. CK19+/GPC3+ HCC has the highest risk of intrahepatic metastasis, microvascular invasion, regional lymph node involvement, and distant metastasis, followed by patients with the CK19−/GPC3+, and then CK19−/GPC3− phenotype. In the current series, although a significant difference in the cumulative RFS rate between CK19+/GPC3+ and CK19−/GPC3+ or CK19−/GPC3−HCC was observed, similar phenomenon between CK19−/GPC3+ and CK19−/GPC3− HCC was not found, which implied that a larger sample size is needed to further investigate the effect of this molecular subtyping method in the risk stratification of HCC patients.

## Supporting Information

S1 Checklist Clinical Studies Checklist. (DOCX)

S1 Table Baseline clinical characteristics of all HCC patients included in the study. (DOCX)
