# Supplementary material for: CK19 and Glypican 3 Expression Profiling in the Prognostic Indication for Patients with HCC after Surgical Resection
Source: PLoS One. 2016 Mar 15;11(3):e0151501. doi: 10.1371/journal.pone.0151501 (PMC4792431; doi:10.1371/journal.pone.0151501)
Supplement: S1 Table — (DOCX) [file pone.0151501.s002.docx]

**S1 Table. Baseline clinical characteristics of all HCC patients included in the study**

| **Variable** |  | **Value** |
| --- | --- | --- |
| **Age, years** | Median | 55.00 |
|  | Range | 17-83 |
|  | Mean±SD | 53.93±10.562 |
| **Male, n(%)** |  | 283(81.79) |
| **Cirrhosis, n(%)** |  | 273(78.90) |
| **TNM staging, n(%)** |  | |
|  | I | 130(37.57) |
|  | II | 119(34.39) |
|  | III | 86(24.86) |
|  | IV | 11(3.18) |
| **Immuno-phenotype , n(%)** |  | |
|  | CK19+/GPC3+ | 69(19.94) |
|  | CK19-/GPC3+ | 224(64.74) |
|  | CK19-/GPC3- | 53(15.32) |
| **Number of tumor nodule, n(%)** |  | |
|  | 1 | 251(72.54) |
|  | 1<n<3 | 95(27.46) |
| **Size of main nodule, n(%)** |  |  |
|  | ≤3cm | 171(49.42) |
|  | >3cm, 5cm≤ | 72(20.81) |
|  | >5cm | 103(29.77) |
| **Histological grading, n(%)** |  | |
|  | poor | 171(49.42) |
|  | moderate | 153(44.22) |
|  | well | 22(6.36) |
| **Microvascular invasion, n(%)** |  | 158(45.66) |
| **Macrovascular invasion, n(%)** |  | 20(5.78) |
| **Histology variation, n(%)** |  | |
|  | Acinar/thin trabecular | 57(16.47) |
|  | Thick trabecular | 243(70.23) |
|  | Compact | 12(3.47) |
|  | scirrhous | 34(9.83) |
| **Perforation of visceral peritoneum, n(%)** |  | 26(7.51) |
| **Regional lymph node involvement, n(%)** |  | 14(4.05) |
| **Distant metastasis, n(%)** |  | 14(4.05) |

HCC, hepatocellular carcinoma; TNM, the TNM (Tumor, Node, Metastasis) staging system
